# Versatile Roles of the Receptor-Like Kinase Feronia in Plant Growth, Development and Host-Pathogen Interaction

**DOI:** 10.3390/ijms21217881

**Published:** 2020-10-23

**Authors:** Dongchao Ji, Tong Chen, Zhanquan Zhang, Boqiang Li, Shiping Tian

**Affiliations:** 1 Key Laboratory of Plant Resources, Institute of Botany, Chinese Academy of Sciences, Beijing 100093, China; jidongchao@ibcas.ac.cn (D.J.); chentong@ibcas.ac.cn (T.C.); zhangzhanquan82@ibcas.ac.cn (Z.Z.); bqli@ibcas.ac.cn (B.L.); 2 University of Chinese Academy of Sciences, Beijing 100049, China; 3 Key Laboratory of Post-Harvest Handling of Fruits, Ministry of Agriculture, Beijing 100093, China

**Keywords:** *Cr*RLK1L, FERONIA, host-pathogen interaction, rapid alkalinization factor

## Abstract

As a member of the *Catharanthus roseus* receptor-like kinase 1-like (*Cr*RLK1L) protein kinase subfamily, FERONIA (FER) has emerged as a versatile player regulating multifaceted functions in growth and development, as well as responses to environmental factors and pathogens. With the concerted efforts of researchers, the molecular mechanism underlying FER-dependent signaling has been gradually elucidated. A number of cellular processes regulated by FER-ligand interactions have been extensively reported, implying cell type-specific mechanisms for FER. Here, we provide a review on the roles of FER in male-female gametophyte recognition, cell elongation, hormonal signaling, stress responses, responses to fungi and bacteria, and present a brief outlook for future efforts.

## 1. Introduction

**Figure 1:**
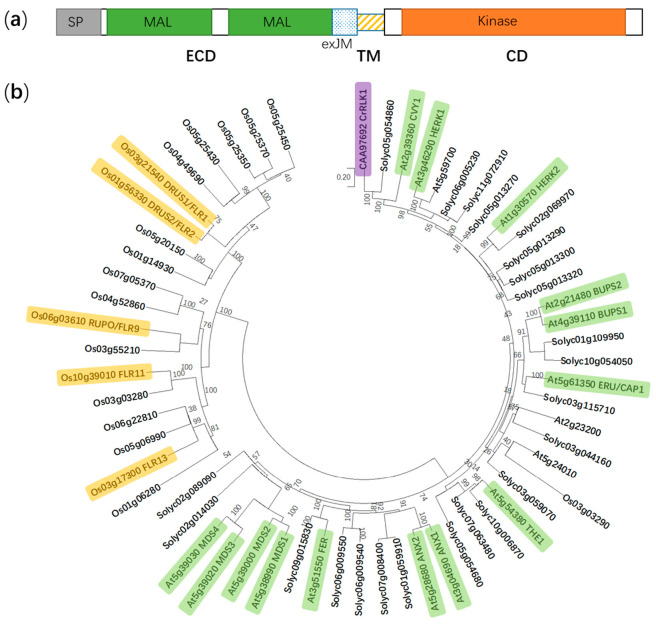
Protein domain structure of FER and a phylogenetic tree of the *Cr*RLK1L subfamily in *Arabidopsis thaliana*, *Solanum lycopersicum*, and *Oryza sativa*. (**a**) FER is composed of an extracellular domain (ECD), a transmembrane domain (TM), and a cytosolic domain (CD). SP, signal peptide; MAL, malectin-like; and exJM, extracellular juxtamembrane region. (**b**) The phylogenetic tree is generated using the neighbor-joining method in MEGA X. Boot-strap values (1000 replicates) are shown for each branch. FER, FERONIA; ANX1 to 2, ANXUR1 to 2; THE1, THESEUS1; ERU/CAP1, ERULUS/(Ca^2+^)_cyt_-ASSOCIATED PROTEIN KINASE; BUPS1 to 2, BUDDHA’S PAPER SEAL 1 to 2; HERK1 to 2, HERCULES1 to 2; CVY1, CURVY1; *Cr*RLK1, *Catharanthus roseus* receptor-like kinase 1; MDS1 to 4, MEDOS1 to 4; FLR1, 2, 9, 11, and 13, FERONIA-LIKE RECEPTOR1, 2, 9, 11, and 13; RUPO, RUPTURED POLLEN TUBE; and DRUS1 to 2, DWARF AND RUNTISH SPIKELET1 to 2. Yellow, green and purple color indicate functionally identified *Cr*RLK1L members in *Oryza sativa*, *A. thaliana* and *Catharanthus roseus*, respectively.

Plants are persistently perceiving, recognizing and transducing extracellular environmental signals, such as light, temperature, nutrients, and pathogens, during their lifetime [1,2,3,4], which have received extensive interest worldwide to dissect the underlying molecular basis. Plant cells usually utilize receptors to interpret diverse signals into cellular responses [5]. Plant receptor-like kinases (RLKs) compose one of the largest subfamilies in membrane proteins, which involve more than 600 members in *Arabidopsis thaliana* and *Solanum lycopersicum*, respectively [6,7]. To date, most of the ligands for these receptor-like kinases have not been identified. A typical RLK contains an extracellular domain, a transmembrane region, and a cytoplasmic kinase domain [8]. According to the differences in the extracellular domains, plant RLKs are divided into several subgroups, including the *Catharanthus roseus* RLK1-like (*Cr*RLK1L) family, the leucine-rich repeat (LRR) RLK family, the LysM RLK family, the proline-rich extensin-like RLK family, and the lectin RLK family [6,9]. Named after the identification of *Cr*RLK1 in *Catharanthus roseus* [10], members in the *Cr*RLK1L family are characterized by one or two malectin-like domains, a transmembrane domain, and an intracellular Ser/Thr kinase domain [9]. The *Cr*RLK1L members always harbor two malectin-like domains, which have been hypothesized to be capable of interacting with cell wall polysaccharides (oligogalacturonides, OGAs) or glycosylated proteins. Currently, a total of 17, 23, and 20 putative *Cr*RLK1L members have been retrieved in the genomes of *A. thaliana*, *S. lycopersicum*, and *Oryza sativa*, respectively [6,11,12], some of which have been functionally identified (Figure 1). Among them, FERONIA (FER) is, above all, the most extensively investigated, which is named from the Etruscan fertility goddess for its originally identified role in fertilization [13,14]. Here, we focus on recent advances in underpinning multifaceted functions of FER (AtFER, unless indicated otherwise) and try to explore further challenges in future studies on this RLK.

## 2. FER is Involved in Multiple Aspects in Plant Growth and Development

### 2.1. FER Mediates Male-Female Gametophyte Recognition During Sexual Reproduction

Precise guidance and penetration of pollen tubes are crucial for successful sexual reproduction, which requires the coordination of extracellular signaling molecules and their receptors [15,16]. The polarized growth of pollen tubes is mainly guided by the chemical inducer LURE [17,18], whereas FER has a dual role in guaranteeing the normal delivery of sperm cells and blocking polyspermy [19,20,21,22]. As early as 2003, Huck et al. isolated a semi-sterile gametophytic mutant affected in female gametophyte development, *feronia* (*fer*), for which the pollen tubes failed to burst and release the sperm cells [14]. Further studies showed that the mutation in *fer* was mapped to At3g51550, which encoded a membrane-resided receptor-like kinase composed of a signal peptide (SP), two extracellular malectin-like domains (MAL), an extracellular juxtamembrane region (exJM), a transmembrane domain (TM), and an intracellular kinase domain (Kinase) (Figure 1A) [19,20,23]. Normal pollen tube growth, arrest, and the release of sperm cells require the FER-dependent signaling pathway in the synergid cell membrane, during which reactive oxygen species (ROS) are key mediators [14,19,22]. LORELEI (LRE) encodes a glycosylphosphatidylinositol (GPI)-anchored protein with a modified eight-cysteine motif (M8CM). In the *lorelei* (*lre*) mutant, pollen tube reception fails in most female gametophytes, whereas the ectopic expression of LRE in pollen tubes could complement the defect in pollen tube reception in *lre* female gametophytes in a non-cell-autonomous manner, which is dependent on FERONIA but nondependent on the GPI anchor. These findings imply that FERONIA and LRE are synergistically involved in pollen tube reception [24]. Moreover, FER interacts with RHO GTPase signaling pathway proteins ROP2 (RHO OF PLANTS 2) and GEF1 (GUANINE EXCHANGE FACTORS 1) to generate ROS burst at the filiform apparatus/synergid cell region, further inducing pollen tube rupture and sperm release [20,21]. Importantly, a point mutation in FER-K565 resulted in the abolishment of its in vitro kinase activity, but it can complement the defect in pollen tube reception of *fer-1*, suggesting that the kinase activity may be not essential for FER function [25].

**Figure 2:**
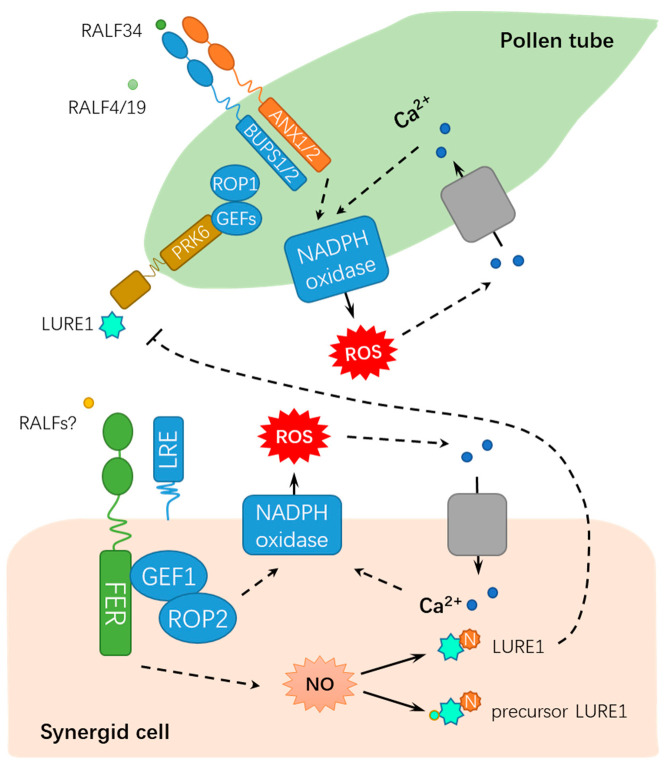
A schematic diagram for the functions of FER in regulating male-female gametophyte recognition during sexual reproduction. FER mediates male-female interaction by ROS, NO, and Ca^2+^ signaling. RALF, rapid alkalinization factor; ROP, RHO OF PLANTS; ROS, reactive oxygen species; GEFs, GUANINE EXCHANGE FACTORS; LRE: LORELEI; PRK6: POLLEN-SPECIFIC RECEPTOR-LIKE KINASE 6; NADPH, nicotinamide adenine dinucleotide phosphate; N, nitrosation; and NO: nitric oxide. Solid lines indicate previously defined signaling pathways. Dash lines indicate transport or speculative steps.

Originally identified to be capable of inducing apoplastic alkalization in a tobacco cell culture, rapid alkalinization factors (RALFs) compose a family of 34 RALF-like genes in *Arabidopsis* [26,27], which are involved in *Cr*RLK1L receptors/FER signaling. Among the *Cr*RLK1L receptors, BUDDHA’S PAPER SEAL l (BUPS1) and BUPS2 (BUPS1/2) and ANXUR1 and ANXUR2 (ANX1/2) have been shown to regulate pollen tube wall stiffness, whereas RALF4 and RALF19 may function in maintaining cell integrity [28,29]. BUPS1/2 and ANX1/2 bind to RALF4 and RALF19 for maintaining pollen tube integrity, whereas RALF34 is capable of binding to BUPS1/2 and ANX1/2 and inducing pollen tube burst at nanomolar concentrations [16]. These findings propose a novel working model by which RALF34 takes over RALF4 and RALF19 during male–female gametophyte communication, thereby triggering pollen tube rupture by deregulating BUPS-ANXUR signaling. In addition to the function for facilitating fertilization, FER also inhibits the entry of supernumerary pollen tubes into the female gametophyte by regulating ovular pectin and nitric oxide [22]. FER maintains de-esterified pectin at the filiform apparatus, whereas pollen tube arrival triggers the FER-dependent accumulation of nitric oxide (NO). Subsequently, NO blocks LURE1 secretion and interaction with its receptor POLLEN-SPECIFIC RECEPTOR-LIKE KINASE 6 (PRK6) by nitrosating both the precursor and mature forms of LURE1, ultimately suppressing pollen tube attraction [22]. Moreover, FER-mediated synergic calcium responses are also involved in the orchestrated programmed cell death events of pollen tubes and receptive synergid, which accurately controls sperm delivery [30]. The dual roles of FER in fertilization demonstrates that FER maintains the homeostasis of the internal female gametophyte environment to fine-tune sperm delivery and block polyspermy, which is crucial for the sexual reproduction of flowering plants. As described above, FER modulates the synergid function by perceiving LURE and modulating ROS and Ca^2+^ signaling, whereas BUPS/ANXUR regulates pollen tube wall stiffness by differentially responding to RALFs, suggesting that these events might be integrated by common ligands of FER and BUPS/ANXUR (e.g., LORELEI, RALFs, or other unknown factors) (Figure 2).

### 2.2. FER Modulates Cellular Expansion and Other Developmental Processes

**Figure 3:**
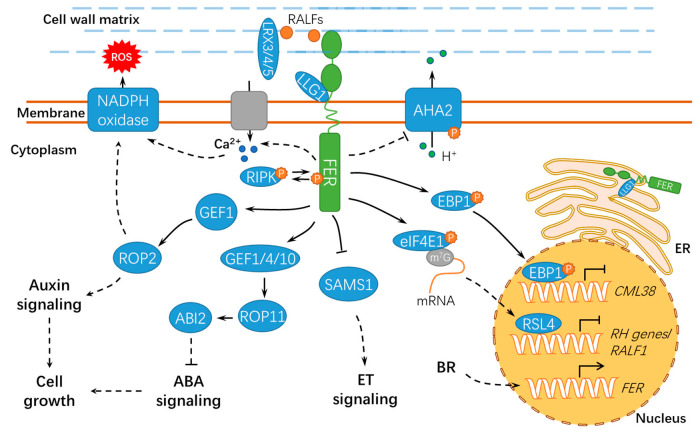
A schematic diagram for the functions of FER in regulating cell growth and hormonal responses. FER regulates cell growth by hormone signaling (such as auxin, ABA, and BR) and RALF signaling. ER, endoplasmic reticulum; ET, ethylene; BR, brassinosteroid; ABA, abscisic acid; ABI2, ABSCISIC ACID INSENSITIVE 2; LLG1, LORELEI-LIKE GLYCOSYLPHOSPHATIDYLINOSITOL-ANCHORED PROTEIN 1; RIPK, RESISTANCE TO *Pseudomonas syringae* pv. *maculicola* 1–INDUCED PROTEIN KINASE; P, phosphate group; m^7^G, 7-methylguanine triphosphate nucleoside (m^7^GpppN); SAMS1, S-ADENOSYLMETHIONINE SYNTHASE1; AHA2, H^+^–ADENOSINE TRIPHOSPHATASE 2; EBP1, ErbB3-binding protein 1; eIF4E1, eukaryotic translation initiation factor 4E 1; RSL4, ROOT HAIR DEFECTIVE 6-LIKE 4; LRX3/4/5, LEUCINE-RICH REPEAT EXTENSIN3/4/5; *CML38*, *CALMODULIN-LIKE PROTEIN 38*; and RH, ROOT HAIR. Solid lines indicate previously defined signaling pathways. Dashed lines indicate transport or speculative steps.

Plants largely depend on cell expansion or elongation for normal growth and development [31]. Root hair is well-known as an excellent model system for investigating cell morphogenesis and polar growth. The mutants of two guanine nucleotide exchange factors for ROPs (RopGEF4 and RopGEF10), *gef4* and *gef10*, displayed decreased cellular ROS levels and compromised root hair initiation and elongation. Further genetic and biochemical evidence indicated that RopGEF4 and RopGEF10 were developmental modulators in FER-mediated root hair growth (Figure 3) but not dependent for the FER-mediated environmental regulation of root hair development [32]. As compared to wild-type *Col*-0, the null mutant *fer-4* shows defects in the development of root hairs and trichomes [20]. FER associates with LORELEI-LIKE GLYCOSYLPHOSPHATIDYLINOSITOL-ANCHORED PROTEIN 1 (LLG1), ROPGEF1 and RAC/ROP2, mediating NADPH oxidase-dependent ROS production, and modulating polarized root hair growth [20,33] (Figure 3). LLG1 acts as a chaperone to maintain normal localization of FER for its proper function in the plasma membrane. Moreover, *fer* and *llg1* mutants share common phenotypes for less ROS accumulation in roots as compared with that in wild-type seedlings. Meanwhile, Huang et al. reported that ROPGEF4 and ROPGEF10 contributed to FER-mediated ROS production during root hair growth [32]. Furthermore, a recent study showed that RALF1-bound FER modulated the mRNA translation and protein synthesis of ROOT HAIR DEFECTIVE 6-LIKE 4 (RSL4) to repress *RALF1* expression and promote root hair tip growth by phosphorylating eukaryotic translation initiation factor 4E 1 (eIF4E1) [34,35].

Notably, RALFs also play important roles in cell expansion (Figure 3). RALF1 interacts with the extracellular domain of the receptor kinase FER, further inhibiting cell elongation by phosphorylating Ser^899^ of H^+^–ADENOSINE TRIPHOSPHATASE 2 (AHA2) to prevent proton transport [31]. Under normal conditions, FER undergoes clathrin-dependent and clathrin-independent endocytosis, and RALF1 substantially stimulated its endocytic internalization [36]. Alternatively, a plant peptide containing the sulfated tyrosine1 (PSY1)-receptor module phosphorylates and activates the plasma membrane H^+^-ATPase, thus displaying antagonistic action to the RALF-FER pathway. PSY1 could trigger a rapid burst of cellular Ca^2+^ and apoplastic alkalinization in *Arabidopsis* roots by activating RALF33 and RALF36. As expected, *fer* mutants did not respond to RALF33 but responded to RALF36 and activated both Ca^2+^ and H^+^ signatures. These results suggest the existence of negative feedback machinery involving different RALF peptides and receptors [37]. Cell elongation mainly relies on the expansion of central vacuoles [38]. Extracellular LEUCINE-RICH REPEAT EXTENSION 3/4/5 (LRX3/4/5) and RALF1 interact with FER, jointly inhibiting vacuolar expansion and preventing cell elongation [38] (Figure 3). Moreover, the RALF1-mediated FER function requires the cytoplasmic kinase RESISTANCE TO *Pseudomonas syringae* pv. *maculicola* 1–INDUCED PROTEIN KINASE (RIPK), and they likely form together a receptor complex downstream of RALF1 [39]. In response to RALF1, FER also facilitates mRNA translation of *ErbB3-binding protein 1* (*EBP1*) and phosphorylates EBP1, resulting in EBP1 accumulation in the nucleus and suppressing *CALMODULIN-LIKE PROTEIN 38* (*CML38*) transcription [40,41]. CML38 serves as a component of the RALF1 signaling pathway via a negative feedback loop to regulate root growth (Figure 3).

**Figure 4:**
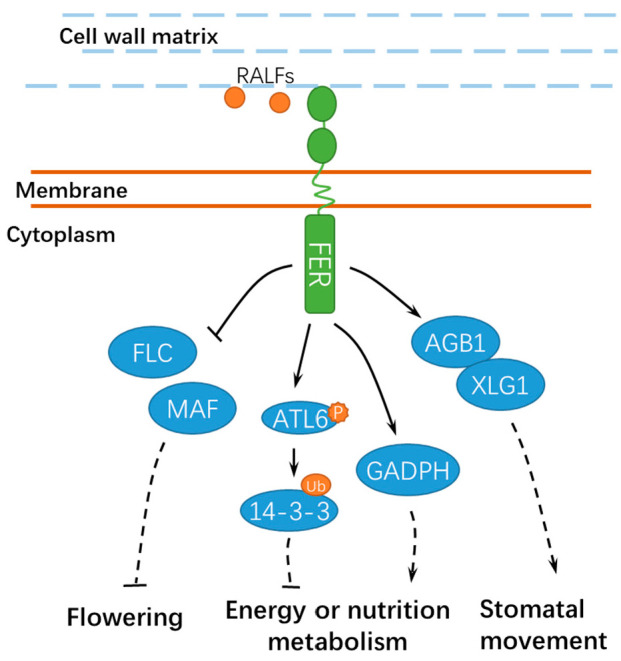
A schematic diagram for the functions of FER in regulating other developmental processes. FER is involved in flowering, energy, or nutrition metabolism and stomatal movement. Ub, ubiquitin; ATL6, *Arabidopsis* Tóxicos en Levadura 6; GADPH, GLYCERALDEHYDE-3-PHOSPHATE DEHYDROGENASE; FLC, FLOWERING LOCUS C; and MAF, MADS AFFECTING FLOWERING. Solid lines indicate previously defined signaling pathways. Dashed lines indicate transport or speculative steps.

Mechanical force and cellular energy metabolism also affect cell growth [42,43] (Figure 4). *fer-4* displayed severely altered Ca^2+^ signature and growth responses upon mechanical stimuli, indicating that FER may be a key regulator of mechanical Ca^2+^ signaling [44]. However, further studies are still required to explore the underlying mechanisms and the specific calcium signaling components involved. FER also contributes to the energy production in glycolysis by interacting with GLYCERALDEHYDE-3-PHOSPHATE DEHYDROGENASEs (GAPDH, GAPC1, and GAPC2), which are responsible for starch accumulation and cell expansion [45]. Moreover, FER-mediated E3 ubiquitin ligase ATL6 (*Arabidopsis* Tóxicos en Levadura 6) phosphorylation mediates the stability of 14-3-3 proteins to control the primary carbon and nitrogen response and plant growth [46]. Notably, FER also interacts with XLG1, a core component in heterotrimeric guanine nucleotide-binding (G) proteins, to regulate stomatal movement (Figure 4). By contrast, RALF1 inhibited stomatal opening and promoted stomatal closure in wild-type seedlings but not in the *agb1* mutant [47]. Importantly, further studies show that FER responses may be either kinase-dependent or kinase-independent, deducing from the phenotypes in rosette leaves of the *fer* complementation lines employing a kinase-dead FERK565R [48], which further confirmed the complexity for the functional mechanisms of FER. A recent study shows that FER also modulates the mRNA alternative splicing and transcript accumulation of *FLOWERING LOCUS C* (*FLC*) and *MADS AFFECTING FLOWERING* (*MAF*) to positively control the flowering time in *Arabidopsis,* which is important for reproductive growth and development [49] (Figure 4). FER and GEF1 (possibly other GEFs) are involved in an undefined signaling pathway to negatively regulate the elongation of integument cells and, ultimately, the seed size in *Arabidopsis* [50].

### 2.3. FER Has Pivotal Roles in Hormone Signaling

Hormones are key regulators for the growth, development, and defenses of plants [9], among which, auxin is well-known for promoting root hair elongation [20,51]. FER functions in a ROPGEF1/ RAC/ROP2 signaling pathway and serves as a modulator for auxin-regulated root hair development [20] (Figure 3). In addition, FER also regulates the F-actin-dependent polar localization of PIN2 and polar auxin transport, thus modulating lateral root branching and the gravitropic response [52]. A recent study showed that FER mediates the root nutating growth via PIN2- and AUX1-mediated auxin transport [53], thus providing new evidence for the correlation of FER to auxin signaling. Moreover, *fer-4* is hypersensitive to abscisic acid (ABA), which can be mimicked by the mutations in GEF1/4/10 or ROP11/ARAC10 (RHO OF PLANTS 11/RAC-LIKE GTP BINDING PROTEIN 10) [54]. These results suggested that FER utilize diverse ROPGEFs in auxin and ABA signaling [54] (Figure 3). In addition, when RALF23 is downregulated in *Arabidopsis* in response to brassinolide (BL) treatment, FER negatively regulates the brassinosteroid (BR) response in hypocotyl growth and promotes the BR response in etiolated seedlings [55]. FER, THESEUS1, and HERCULES1, three RLKs in the *Cr*RLK1L family, are transcriptionally activated by BRs [56]. The *FER* transcripts are downregulated in the BR-insensitive mutant *bri1-5* and upregulated in the gain-of-function mutant *bes1-D* [56]. Microarray studies revealed that these RLKs regulated the expression of a set of genes, including those involved in cell elongation [56], thus implying the existence of gene sets that are commonly and independently regulated by FER and BR to regulate cell elongation. 

In addition, the null mutant *fer-4* showed higher levels of S-adenosylmethionine (SAM, ethylene precursor) and ethylene, whereas the *S-ADENOSYLMETHIONINE SYNTHASE1* (*SAMS1*) and *SAMS2* overexpression lines could mimic the dwarf phenotype of *fer-4* [57]. Coincidently, T-DNA insertion in *FER* (*fer-2* and *fer-3*) led to an enhanced ethylene response in *Arabidopsis*, indicating that FER is a negative regulator of the ethylene response and ethylene is a negative regulator of FER [55]. SAMS is a key enzyme catalyzing the production of SAM in the ethylene biosynthesis pathway [58]. Mao et al. found that SAMS1 and SAMS2 interacted with FER at the plasma membrane in *A. thaliana* [57], repressing SAMS activity and, thus, ethylene production (Figure 3). A similar result was reported for the putative homologs in apples (*Malus* × *domestica*), in which MdFERL6 and MdFERL1 physically interacted with MdSAMS, thus negatively affecting ethylene biosynthesis [59]. However, given the importance of ethylene biosynthesis in climacteric fruit ripening, it is pivotal to examine the specific expression patterns for the putative homologs during the ripening process. Additional efforts are still required to examine whether FER may directly phosphorylate SAMSs to modulate their enzymatic activities. Possibilities for the involvements of other post-translational modifications on SAMSs should not be ruled out. Zermiani and colleagues also reported that ethylene downregulated the transcript abundance of genes involved in ROP-GAP rheostat and further affected apoplastic ROS homeostasis in apple fruits in cold storage, whereas the 1-MCP treatment activated a gene encoding a FERONIA-like kinase [60], suggesting FER homologs in fruit may also have certain regulatory roles in ethylene signaling. In terms of nonclimacteric fruit, ABSCISIC ACID INSENSITIVE 1 (ABI1) negatively regulated the fruit ripening of strawberries (*Fragaria* × *ananassa*) [61]. FaMRLK47, a homolog of AtFER, negatively regulated ABA biosynthesis by interacting with FaABI1, thus inhibiting strawberry fruit ripening [62]. However, it is still uncertain how these putative FER homologs mediate fruit ripening as a kinase or just merely a scaffolding protein.

## 3. Potential Functions of FER in Stress Tolerance

**Figure 5:**
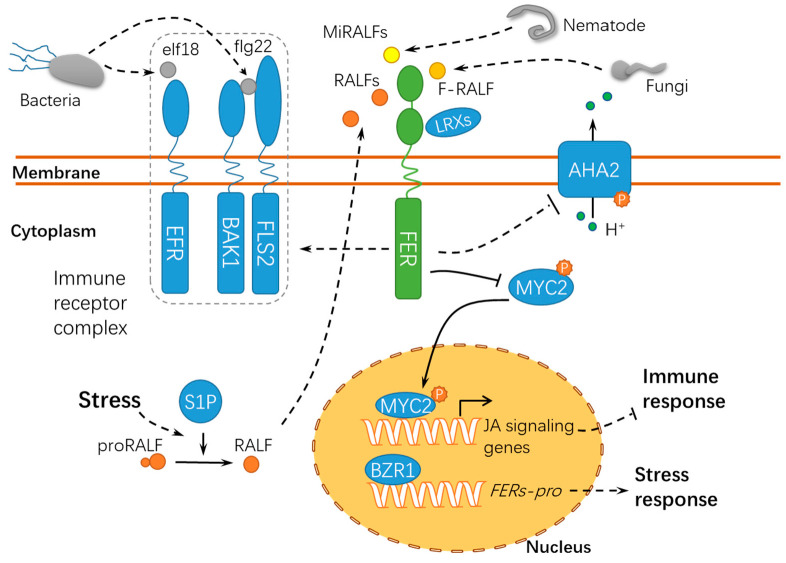
A schematic diagram for the functions of FER in responding to abiotic and biotic stress. FER acts as a scaffold protein to mediate the assembly of the immune receptor complex, during which FER serves as a target of diverse RALFs. proRALF, RALF propeptides; BAK1, BRASSINOSTEROID INSENSITIVE 1–ASSOCIATED KINASE 1; EFR, ELONGATION FACTOR THERMO-UNSTABLE RECEPTOR; elf18, elongation factor Tu peptide (first 18 amino acids); flg22, flagellin epitope 22; FLS2, FLAGELLIN SENSING 2; F-RALF, *Fusarium oxysporum* RALF; MiRALFs: *Meloidogyne incognita* RALFs; S1P, SITE-1 PROTEASE; MYC2, MYELOCYTOMATOSIS PROTEINS 2; LRXs, LEUCINE-RICH REPEAT EXTENSINs; BZR1, BRASSINAZOLE RESISTANT 1; and *FERs-pro*, *FERONIAs promoter*. Solid lines indicate previously defined signaling pathways. Dashed lines indicate transport or speculative steps.

As sessile organisms, plants are confronted with unfavorable environmental conditions, such as high temperatures, salinity, and heavy metals [63,64]. *Arabidopsis* FER and its homologs in other species are also implicated in the responses to stress conditions [63,65,66] (Figure 5).

RLKs may regulate heterotrimeric G-protein signaling in plants [47,67]. The G protein β subunits AGB1 and FER act synergistically to regulate the salt response by stimulating the net K^+^ uptake but inhibiting the net Na^+^ uptake, which requires salt-induced ROS production. AGB1 is involved in inhibiting root-to-shoot Na^+^ translocation under transpiring conditions, whereas FER is mainly involved in inhibiting the net Na^+^ uptake under nontranspiring conditions [67]. During this process, RALF1 enhances the salt toxicity by stimulating Na^+^ accumulation, which is independent of AGB1 and ROS production. Importantly, RALF1 probably inhibits the activity of AHA2 by a FER-dependent mechanism, thus suppressing the Na^+^/H^+^ antiporter activity and leading to salt injuries [67].

*fer-4*, *lrx345* (*leucine-rich repeat extension 345*) triple mutants, and *RALF22/23* overexpression lines are hypersensitive to salt stress, suggesting that these components may function synergistically in response to high salt [65]. LRXs physically interact with RALFs, demonstrating that these proteins function together with FER as a module in salt stress tolerance [65]. High salinity facilitates the cleavage of the RALF22 propeptide by SITE-1 PROTEASE (S1P), resulting in the release of mature RALF22. Mature RALF22/23 peptides interact with FER and then cause its endocytosis [65], and this may represent a different endocytic mechanism as compared to the RALF1-stimulated FER endocytosis [36]. Another report shows that FER can protect seedlings from injuries upon salt stress by modulating cell-specific calcium transients, and this process requires the integration of FER-dependent calcium signaling and cell integrity signaling [66]. The defects in the cell wall integrity of *fer-2* and *fer-4* can be rescued by exogenous calcium and borate treatments, which also facilitate pectin crosslinking possibly by the “egg-box” model [15], suggesting that the perception of cell wall injuries caused by salt stress may directly result from the connection between the extracellular domain of FER and pectin.

Moreover, it is reported that BZR1 (BRASSINAZOLE RESISTANT 1) binds to the promoter region of the genes encoding FER homologs of tomatoes and, thus, regulate the heat stress tolerance in tomatoes by the FER-dependent ROS signaling pathway [63]. FER may be also be involved in metal ion stress [64]; however, the underlying mechanisms are still not elucidated. Recently, it was demonstrated that the interaction of FER with RALF1 resulted in the phosphorylation of the GLYCINE-RICH RNA-BINDING PROTEIN 7 (GRP7) to promote GRP7 nuclear accumulation, thus triggering a rapid and massive RNA alternative splicing response. GRP7 phosphorylation enhanced its mRNA binding ability and its association with the spliceosome component U1-70K, which depends on FER [68].

## 4. FER Has Pivotal Roles in Host-Pathogen Interactions 

Membrane-localized and intracellular immune receptors have crucial roles in the surveillance system recognizing or responding to non-self-components during host-pathogen interactions [69]. Pathogens, particularly necrotrophic pathogens, secrete cell wall hydrolases and form specific infection structures to facilitate host cell invasion, whereas plants have evolved elaborate surveillance machinery to perceive these changes and ward off these invaders [70,71,72]. FER acts as a sensor of cell wall integrity challenged by the host-pathogen interaction and further triggers downstream immune responses in the host cell [73] (Figure 5). Similar to *nta* (*nortia*) mutants, *fer* mutants are insensitive to *Golovinomyces* (*syn. Erysiphe*) *orontii*, indicating that FER may negatively regulate plant immunity to biotrophic pathogens. The *fer*-mediated resistance may be attributed to the activation of the ethylene/jasmonic acid pathways [74]. As compared with the functions of FER in pollen tube guidance and powdery mildew resistance, FER may regulate the susceptibility of plants to symbiotic organisms in the early years of evolution [74]. Another study revealed that Os*FLR2* and Os*FLR11*, two rice *FERONIA-LIKE RECEPTOR* (*FLR*) genes, attenuated the resistance of rice seedlings to *Magnaporthe grisea* by downregulating defense-related genes and suppressing ROS bursts at the penetration sites [75]. It is noteworthy that fungal pathogens secrete peptides with homology to RALF23 [76,77]. During the interaction between host plants and the root-infecting fungus *Fusarium oxysporum*, FER is targeted by a small peptide F-RALF secreted by *F. oxysporum*, leading to extracellular alkalinization and defense response inhibition [76]. This may result from the phosphorylation of PM-resided AHA2 following the F-RALF-FER interaction [31]. The increase in extracellular pH activates a fungal mitogen-activated protein kinase (MAPK), Fmk1, which is indispensable for mycelial growth, plant infection, and the pathogenicity of *F. oxysporum*. Similar RALFs have been found in 26 species of phytopathogenic fungi [77], suggesting an interesting possibility that different RALF peptides may be employed by microorganisms to override plant defenses to facilitate successful infections. Similarly, FER can also bind and respond to the small peptides MiRALF1 and MiRALF3 from *Meloidogyne incognita*, thus modulating the cell expansion and downstream immune responses [78], suggesting that the FER-RALF modules may have similar roles in the responses of plants to developmental cues, pathogen infections, and nematode invasions. As a scaffold protein, FER is recruited into the receptor kinase complex with ELONGATION FACTOR THERMO UNSTABLE RECEPTOR (EFR), FLAGELLIN SENSING 2 (FLS2), and BRASSINOSTEROID INSENSITIVE 1–ASSOCIATED KINASE 1 (BAK1) to initiate immune signaling [79]. *Arabidopsis* SITE-1 PROTEASE (S1P) cleaves the endogenous RALF23 precursor to deliver mature RALF23 and, thus, inhibits the scaffolding function of FER, finally dampening the immune signaling [79]. Meanwhile, FER suppresses the jasmonic acid (JA) and coronatine (COR) signaling pathway by phosphorylating the transcription factor MYC2 (MYELOCYTOMATOSIS PROTEINS 2) [80], thus revealing the RALF23-FER-MYC2 signaling pathway. Xiao et al. reported after X-ray crystallography and a biochemical assay that LLG1 and LLG2 directly bound RALF23 to induce the assembly of RALF23–LLG1–FER and RALF23–LLG2–FER heterocomplexes, respectively, which depends on the conserved N-terminal of RALF23 [81]. Collectively, FER may act as positive or negative regulator in immune responses by binding to RALFs or modulating the immune receptor kinase complex assembly, indicating that FER functions diversely in response to pathogens of different trophic types (Figure 5). It was recently found using high-resolution live cell imaging and single-particle tracking that FER regulates FLS2 dynamics in the nanoscale domains in the plasma membrane, further supporting the hypothesis that FER has crucial roles in recruiting and maintaining the immune receptor complex [82]. Interestingly, immune signaling regulated by FER in response to *P. syringae* is independent of its kinase activity. However, whether this is also applicable to the pathogens of other life strategies remains unknown. Moreover, the *fer-2* mutant incapable of binding pectin is still functional in regulating immune signaling, whereas the wall-associated LRXs are required for this process. Up to now, given the characteristics for FER as a membrane-residing receptor-like kinase, the partners for FER in the host-pathogen interaction still need to be identified.

## 5. Future Outlook

In summary, FER may employ cell type-specific mechanisms by functioning with other protein kinases or simply working as a scaffolding protein to facilitate the recruitment/assembly of signaling complexes [83,84]. In vitro and in vivo kinase assays indicate that FER undergoes autophosphorylation and transphosphorylation [19,40,80]. However, the kinase dead version of FER (FER K565R) influences RALF1-caused root growth inhibition rather than ovule fertilization in *Arabidopsis* [85], indicating that the kinase activity of FER may not account for male-female gametophyte interactions or FER may have cell type-specific functions in different biological contexts. However, deducing from the currently available data, FER may phosphorylate a number of downstream substrates; a high-throughput quantitative phosphor-proteomic analysis may facilitate the identification of kinase substrates and specific phosphorylation sites. Another important aspect in the kinase activity of FER is the dephosphorylation of FER, which has been scarcely reported. When FER is phosphorylated upon RALF1 and ABA stimuli, ABI2 inhibits FER activity by directly dephosphorylating FER [86]. Further studies on the attenuation of FER activity by dephosphorylation merit further investigation in other cellular and physiological contexts.

FER/*Cr*RLK1Ls-RALFs may display opposite effects in different cell types or in response to pathogens of different life strategies. Moreover, different *Cr*RLK1L members may function in coordination in response to diversified environmental stimuli. THESEUS (THE1) functions upstream of GEF4, thus contributing to the resistance of *Arabidopsis* seedlings to the necrotrophic pathogen *Botrytis cinerea* [87], whereas HERK1, THE1, and FER function independent of BR signaling in cell elongation, as *FER-*RNAi seedlings and *herk1 the1* double mutants showed similar dwarf phenotypes during vegetative growth [56]. Therefore, substantial functions of other *Cr*RLK1L members in host-pathogen interactions also deserve further in-depth investigation. The identification of interacting partners and downstream substrates/signaling may greatly facilitate the elucidation of diversified roles of FER in different vital processes, whereas the interaction between extracellular domains of different *Cr*RLK1L members may also become a focus of research interests. In addition to phosphorylation, the FER protein may also have other types of post-translational modifications to exert its functions, which deserves additional work for clarification.

## Abbreviations

FER	FERONIA
ANX1 to 2	ANXUR1 to 2
THE1	THESEUS1
BUPS1 to 2	BUDDHA’S PAPER SEAL 1 to 2
HERK1 to 2	HERCULES1 to 2
CVY1	CURVY1
CrRLK1	Catharanthus roseus receptor-like kinase 1
FLR	FERONIA-LIKE RECEPTOR
RALF	rapid alkalinization factor
ROP	RHO OF PLANTS
ROS	reactive oxygen species
GEFs	GUANINE EXCHANGE FACTORS
LRE	LORELEI
PRK6	POLLEN-SPECIFIC RECEPTOR-LIKE KINASE 6
NADPH	nicotinamide adenine dinucleotide phosphate
NO	nitric oxide
ER	endoplasmic reticulum
ET	ethylene
BR	brassinosteroid
ABA	abscisic acid
ABI2	ABSCISIC ACID INSENSITIVE 2
LLG1	LORELEI-LIKE GLYCOSYLPHOSPHATIDYLINOSITOL-ANCHORED PROTEIN 1
RIPK	RESISTANCE TO Pseudomonas syringae pv. maculicola 1–INDUCED PROTEIN KINASE
SAMS1	S-ADENOSYLMETHIONINE SYNTHASE1
AHA2	H^+^–ADENOSINE TRIPHOSPHATASE 2
EBP1	ErbB3-binding protein 1
eIF4E1	eukaryotic translation initiation factor 4E 1
RSL4	ROOT HAIR DEFECTIVE 6-LIKE 4
LRX3/4/5	LEUCINE-RICH REPEAT EXTENSIN3/4/5
CML38	CALMODULIN-LIKE PROTEIN 38
ATL6	Arabidopsis Tóxicos en Levadura 6
GADPH	GLYCERALDEHYDE-3-PHOSPHATE DEHYDROGENASE
FLC	FLOWERING LOCUS C
MAF	MADS AFFECTING FLOWERING
proRALF	RALF propeptides
BAK1	BRASSINOSTEROID INSENSITIVE 1–ASSOCIATED KINASE 1
EFR	ELONGATION FACTOR THERMO-UNSTABLE RECEPTOR
elf18	elongation factor Tu peptide (first 18 amino acids)
flg22	flagellin epitope 22
FLS2	FLAGELLIN SENSING 2
F-RALF	Fusarium oxysporum RALF
MiRALFs	Meloidogyne incognita RALFs
S1P	SITE-1 PROTEASE
MYC2	MYELOCYTOMATOSIS PROTEINS 2
LRXs	LEUCINE-RICH REPEAT EXTENSINs
BZR1	BRASSINAZOLE RESISTANT 1.
